# Population pharmacokinetic analysis of tepotinib, an oral MET kinase inhibitor, including data from the VISION study

**DOI:** 10.1007/s00280-022-04423-5

**Published:** 2022-04-06

**Authors:** Wenyuan Xiong, Orestis Papasouliotis, E. Niclas Jonsson, Rainer Strotmann, Pascal Girard

**Affiliations:** 1 Merck Institute of Pharmacometrics, Merck KGaA, Lausanne, Switzerland; 2 Pharmetheus AB, Uppsala, Sweden; 3 Quantitative Pharmacology, Merck Healthcare KGaA, Darmstadt, Germany; 4 Present Address: UCB, Bulle, Switzerland

**Keywords:** Tepotinib, Population PK, MET kinase inhibitor, NSCLC

## Abstract

**Purpose:**

Tepotinib is a highly selective, potent, mesenchymal–epithelial transition factor (MET) inhibitor, approved for the treatment of non-small cell lung cancer (NSCLC) harboring *MET* exon 14 skipping. Objectives of this population pharmacokinetic (PK) analysis were to evaluate the dose–exposure relationship of tepotinib and its major circulating metabolite, MSC2571109A, and to identify the intrinsic/extrinsic factors that are predictive of PK variability.

**Methods:**

Data were included from 12 studies in patients with cancer and in healthy participants. A sequential modeling approach was used to analyze the parent and metabolite data, including covariate analyses. Potential associations between observed covariates and PK parameters were illustrated using bootstrap analysis-based forest plots.

**Results:**

A two-compartment model with sequential zero- and first-order absorption, and a first-order elimination from the central compartment, best described the plasma PK of tepotinib in humans across the dose range of 30–1400 mg. The bioavailability of tepotinib was shown to be dose dependent, although bioavailability decreased primarily at doses above the therapeutic dose of 500 mg. The intrinsic factors of race, age, sex, body weight, mild/moderate hepatic impairment and mild/moderate renal impairment, along with the extrinsic factors of opioid analgesic and gefitinib intake, had no relevant effect on tepotinib PK. Tepotinib has a long effective half-life of ~ 32 h.

**Conclusions:**

Tepotinib shows dose proportionality up to at least the therapeutic dose, and time-independent clearance with a profile appropriate for once-daily dosing. None of the covariates identified had a clinically meaningful effect on tepotinib exposure or required dose adjustments.

**Supplementary Information:**

The online version contains supplementary material available at 10.1007/s00280-022-04423-5.

## Introduction

Tepotinib is a highly selective, potent, orally available, reversible, adenosine triphosphate competitive, small molecule mesenchymal–epithelial transition factor (MET) inhibitor, which has been approved in Argentina, Australia, Brazil, Canada, Europe (EU member states, as well as Liechtenstein, Iceland and Norway), Great Britain, Hong Kong, Israel, Japan, Korea, Singapore, Switzerland, Taiwan, and the US for the treatment of non-small cell lung cancer (NSCLC) harboring *MET* exon 14 skipping [1–6]; it is currently undergoing health authority reviews in multiple other countries and regions. The Japanese approval was the first globally for a MET inhibitor [5].

Tepotinib approval is supported by the results of the phase 2 study of tepotinib in patients with NSCLC and confirmed exon 14 skipping mutations (VISION, NCT02864992) [7]. Patients received oral tepotinib 500 mg once daily (QD) over 21-day cycles, and the primary endpoint was the objective response [7]. Tepotinib, administered as monotherapy in the VISION study, was associated with an acceptable and manageable safety profile.

During clinical development, tepotinib was assessed in patients with cancer at doses of 30–1400 mg QD [8–10]. Studies were designed to assess the maximum tolerated dose, recommended phase 2 dose, to compare formulations, and to assess the effects of intrinsic and extrinsic factors [8, 11, 12]. Several different capsule formulations (CFs) and tablet formulations (TFs) have been used during clinical development, aimed at increasing bioavailability and reducing pharmacokinetic (PK) variability. The initial CF formulation, CF1, contained non-micronized material, whereas all subsequent CFs and TFs used micronized material. CF1 was only used for part of the first-in-human study, with doses up to 230 mg [8]. This study also investigated another capsule formulation, CF2, at doses up to 1400 mg, as well as a tablet formation, TF1, at 500 and 1000 mg. The VISION study was conducted with TF2, another micronized tablet formulation with a dose of 500 mg (450 mg active moiety) [7]. The commercial formulation is TF3, a micronized tablet formulation with a dose strength of 250 mg, which has shown bioequivalence to TF2 (manuscript in preparation).

Tepotinib is absorbed after oral administration with a time to maximum concentration (T_max_) at steady state under fed conditions of approximately 8 h, and a terminal elimination half-life of approximately 32 h [1]. Tepotinib is a low-solubility drug [13] but has a high absolute bioavailability of 72% [14]. It is extensively cleared by the liver, predominantly via biliary clearance of unchanged drug, but is also metabolized via multiple pathways. Based on in vitro studies and human mass balance data, no metabolic pathway is dominant, and no pathway comprises more than 25% of the administered dose. Therefore, the interaction potential with co-administered drugs that interact with drug-metabolizing enzymes is considered to be low. The major circulating metabolite is MSC2571109A (R-enantiomer of M506), which contributed 41% of total circulating radioactivity (with tepotinib contributing 55% of the total radioactivity). Negligible amounts of this metabolite were excreted [14]. MSC2571109A is not thought to contribute to the efficacy of tepotinib, based on preclinical PK/efficacy and clinical PK profiling [15].

The objectives of this population PK analysis were to evaluate the dose–exposure relationship of tepotinib and its major circulating metabolite, MSC2571109A, in the overall population, and to identify the intrinsic and extrinsic factors that are predictive of PK variability.

## Methods

### Analysis set

The population PK analysis was based on data collected from 12 studies: five completed studies in patients with cancer, six completed studies in healthy participants, and the pivotal study (VISION) in patients with NSCLC harboring *MET* exon 14 skipping that was ongoing at the time of the analysis. An overview of these studies is provided in Table 1.

**Table 1:** Studies included in the population PK analysis

Study code	Study description	Dose regimen and formulation	No. participants treated with tepotinib and with evaluable PK	PK sampling used in modeling project
001 (NCT01014936) [8]	Phase 1, first-in-human, dose-escalation study	Regimen 1: 30–230 mg CF1, 30–400 mg CF2; QD for 2 weeks, no treatment for 1 week Regimen 2: 30–115 mg CF1, 30–315 mg CF2; TIW for 3 weeks Regimen 3: 300–1400 mg CF2, 500 mg TF1; QD for 3 weeks All regimens at least one 21-day cycle Fed (standard breakfast): CF1, CF2, TF1 Fasted: CF1	149 patients with advanced solid tumors	Rich sampling: 0–72 h post-last dose
002	Phase 1 study to investigate relative bioavailability and food effect	TF1: 30 mg single dose, fed or fasted TF1 or CF2: 30 mg single dose, fed	28 healthy participants	Rich sampling: 0–3 weeks post-dose
003 (NCT01832506) [10]	Phase 1 Japanese dose-escalation study	CF2 fed (standard breakfast): 215, 300 or 500 mg QD over 21-day cycles	12 Japanese patients with solid tumors	Rich sampling: 0–24 h post-dose
004 (NCT01988493) [23]	Phase 1b/2 study to compare tepotinib monotherapy vs sorafenib	TF1 fed (standard breakfast): 300, 500 or 1000 mg QD over 21-day cycles	Phase 1b: 27 Asian patients with BCLC Stage B or C HCC and Child–Pugh class A liver function Phase 2: 45 Asian patients with MET+ BCLC Stage B or C HCC and Child–Pugh class A liver function	Phase 1b: Rich sampling 0–24 h post-dose Phase 2: Sparse sampling
005 (NCT02115373) [24, 25]	Phase 1b/2 study to evaluate tepotinib monotherapy in patients who had failed sorafenib	TF1 fed (standard breakfast): 300 or 500 mg QD over 21-day cycles	Phase 1b: 17 patients with advanced HCC and Child–Pugh class A liver function pretreated with sorafenib Phase 2: 49 patients with MET overexpression, advanced HCC and Child–Pugh class A liver function pretreated with sorafenib	Phase 1b: Rich sampling 0–24 h post-dose Phase 2: Sparse sampling 0–24 h post-dose
006 (NCT01982955) [9]	Phase 1b/2 study to compare combined tepotinib + gefitinib vs chemotherapy as second-line therapy	Phase 1b: TF1 fed (standard breakfast): 300 or 500 mg QD over 21-day cycles with gefitinib Phase 2: TF1 fed (standard breakfast): 300 or 500 mg QD over 21-day cycles with gefitinib or pemetrexed + cisplatin/carboplatin	Phase 1b: 18 patients with MET + advanced/metastatic NSCLC Phase 2: 45 patients with MET + advanced/metastatic NSCLC and resistance to 1st/2nd generation EGFR-TKI	Phase 1b: Rich sampling 0–24 h post-dose Phase 2: Sparse sampling
007 [14]	Phase 1 study to investigate absolute and relative bioavailability, mass balance and metabolite profile	Single dose, fed (high-fat breakfast) Mass balance and metabolite profile: CF3 ^14^C-labeled (498 mg) Absolute bioavailability: TF1 (500 mg) + IV ^14^C-labeled microtracer dose Relative bioavailability: oral solution, TF1 and TF1 (100 mg)	27 healthy male participants	Rich sampling: Mass balance: 0–3.5 weeks post-dose Absolute bioavailability: 0–2 weeks post-dose Relative bioavailability: 0–3 weeks post-dose
012 (NCT03021642)	Phase 1 study to investigate relative bioavailability of two film-coated tablet formulations	TF1 and TF2: 500 mg single dose, fed (high-fat breakfast)	24 healthy participants	Rich sampling 0–3 weeks post-dose
0022 (NCT02864992) [7]	Phase 2 study to investigate tepotinib in advanced stage/metastatic NSCLC (VISION)	TF2 and TF3 fed (standard breakfast): 500 mg QD over 21-day cycles	99 patients with *MET* exon 14 skipping alterations, including 5 with concomitant *MET* amplification	Sparse sampling 0–4 h post-dose
0028 (NCT03546608) [26]	Phase 1 study to investigate the effect of various degrees of hepatic impairment on tepotinib	TF2 fed: 500 mg single dose	6 healthy participants (normal hepatic function) 6 participants with Child–Pugh class A (mild) hepatic impairment 6 participants with Child–Pugh class B (moderate) hepatic impairment	Rich sampling 0–2 weeks in healthy participants and 0–3 weeks in participants with hepatic impairment
0039 (NCT03531762) [11, 27]	Phase 1 study to investigate the effect of omeprazole on tepotinib PK	TF2 fed (standard breakfast) or fasted, 500 mg single dose	12 healthy participants	Rich sampling 0–144 h post-dose
0044 (NCT03629223) [11, 28]	Phase 1 study to investigate bioequivalence of TF3 compared to TF2 (part A) and effect of food on TF2 (part B) and TF3 (part C)	Part A: TF2 then TF3 or TF3 then TF2 500 mg single dose, fasted Part B: TF2 500 mg single dose, fed or fasted Part C: TF3 500 mg single dose, fed or fasted	65 healthy participants	Rich sampling 0–168 h post-dose

All doses are oral unless stated otherwise

*BCLC* Barcelona Clinic liver cancer, *CF* capsule formulation, *EGFR* epidermal growth factor receptor, *HCC* hepatocellular carcinoma, *IV* intravenous, *MET* mesenchymal–epithelial transition factor, *MET* + *MET* exon 14 diagnostic-positive status, *NSCLC* non-small cell lung cancer, *PK* pharmacokinetics, *QD* once daily, *TF* tablet formulation, *TIW* 3 times a week, *TKI* tyrosine kinase inhibitor

All tepotinib and metabolite concentrations were quantified using validated LC–MS/MS methods with lower limits of quantitation (LLOQ) of 5 ng/mL (0.168 ng/mL for studies 001 and 002), and 0.5 ng/mL for tepotinib and MSC2571109A, respectively.

All patients with at least one PK observation above the LLOQ were included in the analysis. Data below the LLOQ were excluded.

### Population PK base model development

A sequential modeling approach was applied to analyze the parent and metabolite data. The tepotinib model was developed first, and the final tepotinib model was extended to characterize the MSC2571109A data, while keeping the individual parameters of the tepotinib model fixed.

Informed by graphical analysis, the following structural and statistical models were evaluated for tepotinib and the metabolite: absorption models for tepotinib, including first-order and sequential zero- and first-order absorption models, and disposition models for tepotinib and MSC2571109A, including one- and two-compartmental disposition models with first-order elimination from the central compartments. Interindividual random variability (IIV) was added to all relevant PK parameters, according to a lognormal distribution with standard deviation ω.

### Covariate model development

A stepwise approach was utilized where an initial structural covariate model was established from the tepotinib base model, followed by a broader scope of covariate searching. Structural covariates per design that were known to impact the absorption of tepotinib were included into the base model after confirmation by graphical exploration and testing their relationship to the absorption-related parameters in a stepwise covariate model building procedure (SCM). This step included food status, formulation, and oral dose level.

In the second step, the remaining covariates were further evaluated, including demographics (age, body weight, race, and sex), patient characteristics (disease status, i.e., patient or healthy participant, tumor type, National Cancer Institute Organ Dysfunction Working Group [NCI-ODWG] classification of liver impairment [16], and baseline tumor burden), laboratory values (serum albumin, total bilirubin, alkaline phosphatase, international normalized ratio, and total protein), and co-medications (opioid analgesics and gefitinib). Since the phase 1 cross-over study 0039 did not show a relevant effect of concomitant omeprazole administration [11], co-administration of proton pump inhibitors was not investigated as a covariate in the PopPK analysis.

Tumor types included hepatocellular carcinoma (HCC), NSCLC, renal cell carcinoma, gastroesophageal cancer, colorectal cancer, and the remaining cancer categories (head/neck, breast, prostate, pancreas, and other solid tumors) combined.

The oral dose level was tested for its effect on apparent clearance (CL) of tepotinib. Estimated glomerular filtration rate (eGFR; calculated using the Modification of Diet in Renal Disease [MDRD] method [17]) was tested for its effect on apparent CL of tepotinib and MSC2571109A. The remaining covariates were tested for their effects on all PK parameters of tepotinib and MSC2571109A. The baseline value was assessed for most of the covariates, except for co-medication with μ-opioids, oral dose level and formulation, which were evaluated as time-varying covariates. Missing covariates were imputed with the population median value or most common value for continuous and categorical covariates, respectively. Disease-related covariates such as tumor size at baseline, were imputed with 0 for healthy participants and the median value among patients for patients.

SCM was applied in the structural covariate model building step, followed by an adaptive scope reduction (ASR) SCM in the following covariate searching step to reduce run times [18]. For the SCM procedures, the forward selection *p* value was set to 0.01, and the backward elimination *p* value to 0.001. The ASR threshold *p* value was set to the same as the forward *p* value of 0.01.

Continuous covariate relationships were coded as power models (Formula 1), and categorical covariates were coded as a fractional difference to the most common category (Formula 2). An alternative ‘hockey-stick’ parameterization (Formula 3) was tested for dose as continuous covariate on bioavailability of the parent drug (Par).

1 $${\text{ParCov}}_{\text{m}} = \left( {\frac{{{\text{Cov}}}}{{{\text{Cov}}_{{{\text{ref}}}} }}} \right)^{{\theta} {\text{m}}}$$

2 $${\text{ParCov}}_{m} = \left\{ \begin{gathered} 1\;\;\;\;\;\;\;\;\;{\text{if}}\;{\text{Cov}} = {\text{Cov}}_{{{\text{ref}}}} \hfill \\ 1 + \theta_{m} \;\;\;{\text{if}}\;{\text{Cov}} \ne {\text{Cov}}_{{{\text{ref}}}} \hfill \\ \end{gathered} \right.$$

3 $${\text{ParCov}}_{m} = \left\{ \begin{gathered} 1 + \theta_{m1} \cdot \left( {{\text{Cov-Cov}}_{{{\text{ref}}}} } \right)\;\;\;\;\;\;\;\;\;{\text{if}}\;{\text{Cov}} \le {\text{Cov}}_{{{\text{ref}}}} \hfill \\ 1 + \theta_{m2} \cdot \left( {{\text{Cov-Cov}}_{{{\text{ref}}}} } \right)\;\;\;\;\;\;\;\;\;{\text{if}}\;{\text{Cov}}\;{ > }\;{\text{Cov}}_{{{\text{ref}}}} \hfill \\ \end{gathered} \right.$$

ParCov_m_ is covariate m (Cov_m_) for the parent drug and Cov_ref_ is a reference covariate value for Cov_m_, to which the covariate model is normalized (usually the median or mode).

The IIV covariance structure and residual unexplained variability (RUV) terms were re-assessed to establish the final covariate model.

### Model selection and qualification

Model selection and qualification was based on the changes in the objective function value (OFV) provided by NONMEM and the visual inspection of graphical diagnostics, including goodness-of-fit plots and visual predictive checks (VPCs). For a more complicated model to be retained, it had to provide a significant improvement over the contending model (*p* < 0.05 hierarchical models), and provide plausible parameter estimates not associated with excessively high relative standard errors. It also had to demonstrate improvement in the graphical diagnostics and not result in a high (> 1000) condition number of the correlation matrix, indicating model instability [19].

The population PK analyses were performed using NONMEM version 7.3.0. Data management and further processing of NONMEM output were performed using R version 3.3.3. VPCs and covariate model building were run on PsN version 4.4.8.

### Forest plot

The association or lack of association between the observed covariates and model-derived secondary PK parameters [e.g., area under the concentration–time curve over the dosing interval at steady state (AUC_τ,ss_)] for patients with cancer was illustrated using bootstrap analysis-based forest plots (see ESM 1 for method details). In the bootstrap analysis, *N* = 100 new data sets were generated with replacement from the analysis data set. The sampling was stratified by study, and the final models were fit to each of the resampled data sets. In the bootstrap for MSC2571109A, the parameter estimation was conditioned on the individual parameter estimates from the final tepotinib model. For categorical covariates, the mean and its 5th and 95th percentiles of PK parameters for each category were computed. For continuous covariates, the mean and its 5th and 95th percentiles of PK parameters, corresponding to the 0–5th and 95–100th percentile of the covariate, were computed. The impact of the covariates in the forest plots were presented on a relative scale. The reference value was the arithmetic mean of the corresponding PK parameter.

## Results

### Summary of analysis data

A total of 10,788 tepotinib concentrations from 613 study participants and 7197 MSC2571109A concentrations from 464 participants were included in the analysis. Key characteristics of the tepotinib and MSC2571109A analysis data sets are provided in Table 2. Concentrations of MSC2571109A were only measured after its discovery in the mass balance study [13], while the clinical development was ongoing; hence, there are fewer observation records available for MSC2571109A than tepotinib.

**Table 2:** Key characteristics of the tepotinib and MSC2571109A analysis data sets

	Tepotinib	MSC2571109A
Number of participants	613	464
Number of samples	10,788	7197
Development phases	1, 1b, 2	1, 1b, 2
Age, years	58 (18–89)^a^	60 (18–89)^a^
Weight, kg	72.0 (35.5–136)^a^	72.0 (35.5–136)^a^
Sex		
Male	439	334
Female	174	130
Participant type		
Patients with cancer	438	344
Without cancer	175	120
Race		
Caucasian	362	239
Japanese	28	28
Other East Asian	145	143
African origin	17	8
Hispanic	25	14
Other/missing	36	32
Dose levels	30 to 1400 mg/day	215 to 1000 mg/day
Route of administration and formulation	Capsules (oral): non-micronized (CF1) and micronized (CF2)	Capsules (oral): micronized (CF2)
	Tablets (oral): micronized (TF1, TF2), finely micronized (TF1*), marketed formulation (TF3)	Tablets (oral): micronized (TF1, TF2), marketed formulation (TF3)
Regimens	Once daily, 3 times per week, single dose	Once daily, 3 times per week, single dose
Dosing in relation to meals	Fasted, fed standard breakfast, fed high-fat/high-calorie breakfast	Fasted, fed standard breakfast, fed high-fat/high-calorie breakfast

TF1* - this is the version of TF1 that contains finely micronized drug (as opposed to TF1 which contains justmicronized drug)

^a^Median (range)

The analysis included 591 participants with an evaluable baseline eGFR (mean 99.8 mL/min/1.73 m^2^, standard deviation 26.2 mL/min/1.73 m^2^, range 39.4 to 236 mL/min/1.73 m^2^), of which 24 were classified as having moderate renal impairment (eGFR ≥ 30 and < 60 mL/min/1.73 m^2^) and contributed 390 tepotinib PK observations (3.6% to the total data set).

A total of 146 participants were classed as having mild hepatic impairment (contributing 18.3% of the total number of tepotinib PK observations) and 16 were classed as having moderate hepatic impairment (contributing 1.5% of the total number of tepotinib PK observations), according to the NCI-ODWG criteria for hepatic dysfunction. Only three participants were classed as having severe hepatic impairment (contributing 0.6% of the total number of tepotinib PK observations).

The fraction of participants with missing covariate values ranged between 0 and 18%. The three covariates with the largest fraction of missing values were hepatitis B virus/hepatitis C virus (HBV/HCV) status at screening, serum-albumin and total protein at baseline, with 18%, 17% and 13% missing values, respectively.

### Tepotinib population PK model

**Fig. 1:**
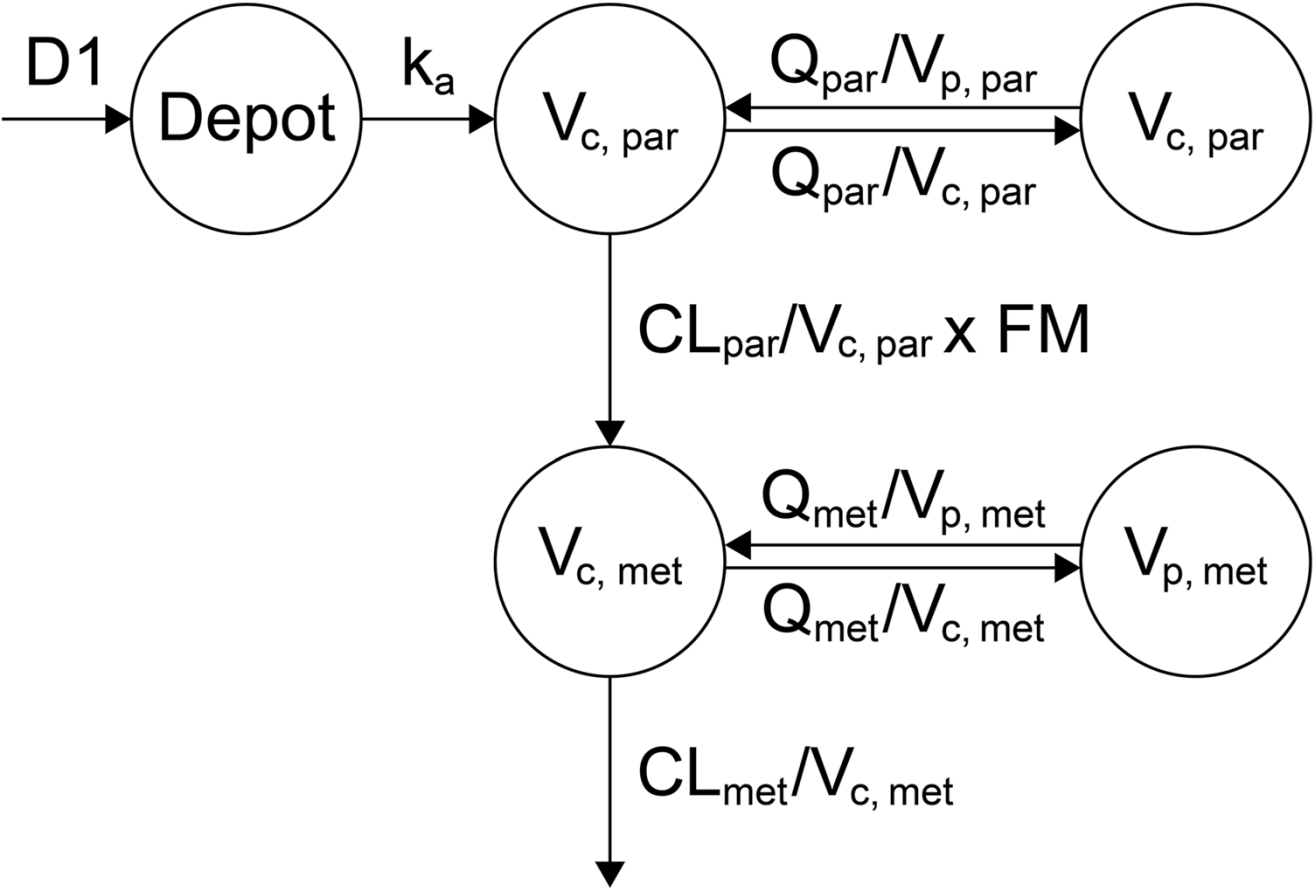
Illustration of the base population pharmacokinetics model for tepotinib and MSC2571109A. *CL* clearance, *D*_*1*_ zero-order absorption duration, *FM* fraction of tepotinib metabolized to MSC2571109A, *k*_*a*_ first-order absorption rate constant, *met* metabolite, *par* parent, *Q* inter-compartmental clearance, *V*_*c*_ central volume of distribution, *V*_*p*_ peripheral volume of distribution

A two-compartment model with sequential zero- and first-order absorption, and a first-order elimination from the central compartment, with an additive residual error on the log scale, best described the plasma PK of tepotinib in humans across the dose range of 30–1400 mg (Fig. 1). The IIV terms are included on CL_par_, first-order absorption rate constant (k_a_), zero-order absorption duration (D1), and bioavailability of parent drug (F_par_).

Apparent CL for tepotinib was estimated at 20.4 L/h, with an IIV of 33.5%. The relative absorption bioavailability was fixed to 1, with an estimated IIV of 28.3%. Tepotinib has a long effective half-life of ~ 32 h.

Several covariates were found to have a statistically significant influence on tepotinib PK parameters based on the ASR SCM procedure; all are listed in Table 3. Tepotinib exposure was significantly reduced after administration of CF1 (65.6% reduction of F_par_, 44.2% reduction of k_a_), and after fasting (20.9% reduction of F_par_, 56.1% reduction of k_a_), compared with a typical participant taking TF2 with a non-high-fat meal.

**Table 3:** Parameter estimates of the final tepotinib and MSC2571109A population PK model

Variable	Final model for tepotinib		
	Value	RSE (%)	SHR (%)
Tepotinib			
CL_par_/F^a^ (L/h)	20.4	2.07	
V_c,par_/F^a^ (L)	1020	2.00	
k_a_ (h^−1)^	0.278	6.16	
Q_par_/F^a^ (L/h)	1.32	4.22	
V_p,par_/F^a^ (L)	1180	16.6	
D1 (h)	4.09	5.34	
Relative F_par_ (CV)	1.00	(FIX)	
Fasting state covariate on D1	–0.370	5.19	
DOSE covariate on F_par_ (/100 mg)	–0.0412	9.71	
Fasting state covariate on F_par_	–0.209	4.94	
High-fat meal covariate on F_par_	0.320	5.96	
CF1 covariate on F_par_	–0.656	7.67	
TF3 covariate on F_par_	0.154	7.08	
Fasting state covariate on k_a_	–0.561	2.63	
CF1 covariate on k_a_	–0.442	15.1	
TF1 covariate on k_a_	0.305	6.35	
TF1* covariate on k_a_	0.674	10.2	
eGFR at baseline covariate on CL_par_/F	0.199	23.5	
Hepatocellular carcinoma covariate on CL_par_/F	0.130	44.2	
Colorectal cancer covariate on CL_par_/F	–0.281	16.4	
μ-Opioids covariate on CL_par_/F	–0.167	10.1	
NCI-ODG class > 0 covariate (liver dysfunction) on D1	–0.332	6.09	
Body weight at baseline covariate on F_par_	–0.475	15.4	
NCI-ODG class > 0 covariate on F_par_	–0.0729	16.7	
INR at baseline covariate on Q_par_/F	3.81	10.5	
Serum albumin at baseline covariate on Q_par_/F	4.14		
Age covariate on V_c,par_/F	0.219	14.7 10.9	
Non-small cell lung cancer covariate on V_c_,_par_/F	–0.232	13.2	
Patient/participant covariate on V_p_,_par_/F	–0.810	4.52	
Study MS200095-0028 covariate on CL_par_/F	–0.115	22.4	
IIV CL_par_ (CV)	0.335	4.57	22.7
IIV k_a_ (CV)	0.653	5.86	29.4
IIV D1 (CV)	0.652	4.98	24.1
IIV F_par_ (CV)	0.283	5.78	28.6
IIV F_par_ for CF1 (CV)	0.713	10.8	2.40
IIV CL_par_ for healthy participant (CV)	0.128	7.64	12.7
IIV F_par_ for healthy participant (CV)	0.188	6.38	12.7
Prop. RUV (CV)	0.337	0.351	6.39
MSC2571109A			
CL_met_^a^ (L/h)	40.2	2.40	
V_c, met_^a^ (L)	131	5.04	
Q_met_^a^ (L/h)	106	5.98	
V_p, met_^a^ (L)	152	2.90	
eGFR at baseline covariate on CL_met_	0.311	24.2	
Body weight at baseline covariate on CL_met_	–0.696	11.7	
Non-small cell lung cancer covariate on CL_met_	0.498	17.2	
Hepatocellular carcinoma covariate on FM	–0.398	5.77	
East Asian on Q_met_	1.40	35.5	
Patient/participant covariate on V_p,met_	2.31	2.17	
NCI-ODG class > 0 covariate on V_c.met_	0.520	9.46	
IIV CL_met_ (CV)	0.536	2.30	6.77
IIV V_c,met_ (CV)	0.859	4.11	15.9
IIV Q_met_ (CV)	0.791	13.6	56.3
IIV V_p,met_ (CV)	0.248	14.1	60.8
IIV CL_met_ for healthy participants (CV)	0.255	6.25	2.28
Pro. RUV (CV)	0.298	0.419	4.17

The reference participant for the parameter estimates in Table 3 was a 59-year-old, non-East Asian patient weighing 72 kg with an EGFR of 97.3 mL/min/1.73 m^2^, an INR of 1.06, with a NCI-ODG classification at baseline of 0, a baseline serum albumin level of 4 g/L, with no concomitant opioid administration, treated with 500 mg of tepotinib TF2 while fed a non-high-fat meal

*CF1* capsule formulation 1 (non-micronized), *CL* clearance, *CL/F* apparent clearance, *COV* covariance, *CV* coefficient of variation, *D*_1_ zero-order absorption duration, *eGFR* estimated glomerular filtration rate, *F* bioavailability, *FM* fraction of tepotinib metabolized to MSC2571109A, *IIV* inter-individual variability, *INR* international normalized ratio of prothrombin time, *k*_*a*_ first-order absorption rate constant, *met* metabolite, *NCI-ODG* National Cancer Institute Organ Dysfunction Group Class, *OFV* objective function value, *Q* inter-compartmental clearance, *par* parent, *PK* pharmacokinetics, *RSE* relative standard error, *RUV* residual unexplained variability, *SHR* shrinkage, *TF1/3* tablet formulations, containing micronized drug substance, TF1* tablet formulation 1, containing finely micronized drug substance, *V*_*c*_ central volume of distribution, *V*_*p*_ peripheral volume of distribution

^a^Multiplied by a factor of 0.9 to correct for the salt to base molar weight ratio

**Fig. 2:**
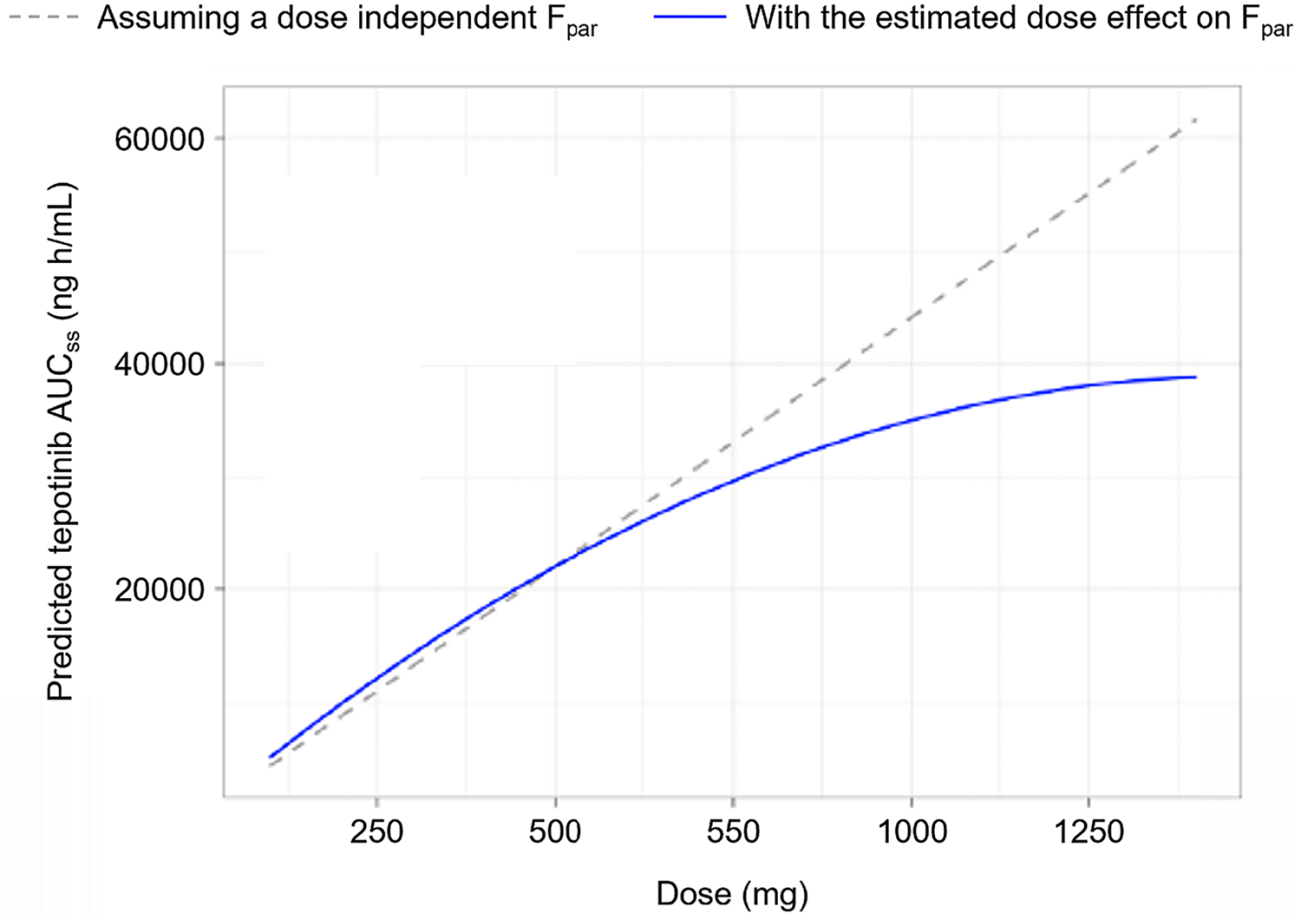
Predicted tepotinib AUC_ss_ versus dose, with and without the estimated dose effect on F_par_. The blue line represents the relationship between AUC_ss_ and dose according to the final tepotinib model, while the grey, dashed line displays the theoretical relationship between tepotinib AUC_ss_ and dose if the dose had no impact on F_par_. *AUC*_*ss*_ area under the curve at steady state, *par* parent, *eGFR* estimated glomerular filtration rate, *INR* international normalized ratio, *NSCLC* non-small cell lung cancer, *QD* once daily, *TF* tablet formulation. Note: The prediction is for a typical patient with NSCLC (59 years, 72 kg, serum albumin = 40 g/L, eGFR = 97.28 mL/min/1.73 m^2^, INR = 1.06) receiving 500 mg QD tepotinib TF3 with food

The bioavailability of tepotinib was shown to be dose-dependent, with decreasing bioavailability at high doses (Fig. 2). The model-predicted effect of tepotinib AUC_τ,ss_ is illustrated in Fig. 2 and indicates a lack of relevant deviation from dose linearity at tepotinib doses up to 500 mg. However, relative bioavailability for the supratherapeutic tepotinib dose of 1000 mg is 0.79 compared with the F_par_ for a dose of 500 mg.

Liver dysfunction (defined by NCI-ODWG) slightly reduced tepotinib bioavailability (7.29%) after oral administration, causing decreased total exposure in participants with liver dysfunction or cirrhosis. The apparent CL of tepotinib was found to be positively correlated to eGFR, but with a seemingly limited influence on exposure (eGFRs of 30, 60, 80 to 110 mL/min/1.73 m^2^ for a typical individual receiving 500 mg of tepotinib led to CL_par_ values of 16.2, 18.6, 19.7 and 20.9 L/h, respectively). Intake of opioid analgesics caused a 16.7% reduction in apparent CL of tepotinib, but the influence on tepotinib AUC_τ,ss_ also appeared to be limited. No statistically significant influence of gefitinib co-administration on tepotinib PK was identified. Refer to ESM 12 for observed vs. predicted tepotinib plasma concentrations. The equations for the final pop PK model are available in ESM 13.

### MSC2571109A population PK model

The plasma PK of MSC2571109A was best described by a two-compartment model that includes input from the central compartment of the tepotinib model, scaled by fraction of tepotinib metabolized to MSC2571109A and a first-order elimination from the central compartment.

The fraction metabolized (FM) is unknown and unidentifiable from the current data, thus this parameter was fixed to 1 but the associated IIV was estimated. The IIV terms are also included on apparent CL of metabolite (CL_met_), central volume of metabolite distribution (V_c,met_), inter-compartmental metabolite clearance (Q_met_) and peripheral volume of metabolite distribution (V_p,met_).

In the covariate modeling step, several covariates were identified to significantly influence MSC2571109A PK parameters (Table 3). Apparent CL was positively correlated with eGFR and negatively correlated with body weight.

Values of derived secondary PK parameters for patients with cancer receiving the clinical dose of tepotinib TF3 500 mg/day with food or a reduced dose level of 250 mg/day were also estimated using a bootstrap analysis of the final population model (see ESM 2).

### Model qualification

Goodness-of-fit plots for the final models of tepotinib (ESM 3 and ESM 4) and MSC2571109A (ESM 5 and ESM 6) indicated that these final models well described the plasma concentrations of both the parent and metabolite. Tepotinib PK appeared to be time-independent, i.e., the PK after a single dose was predictive of the PK at steady state.

The prediction-corrected VPC showed a strong agreement for the observed and model-predicted median PK profiles. Plots of the individual random effects on tepotinib apparent CL (ESM 7 and ESM 8) and bioavailability (ESM 9 and ESM 10), estimated with the base and final models and plotted against individual covariates, confirm that including covariates in the tepotinib model removed the trend in the random effect versus covariates observed with the base model. Model diagnostic plots demonstrated a strong model performance, supporting its usability to derive individual predictions of exposure.

### Model-based simulation and forest plots

**Fig. 3:**
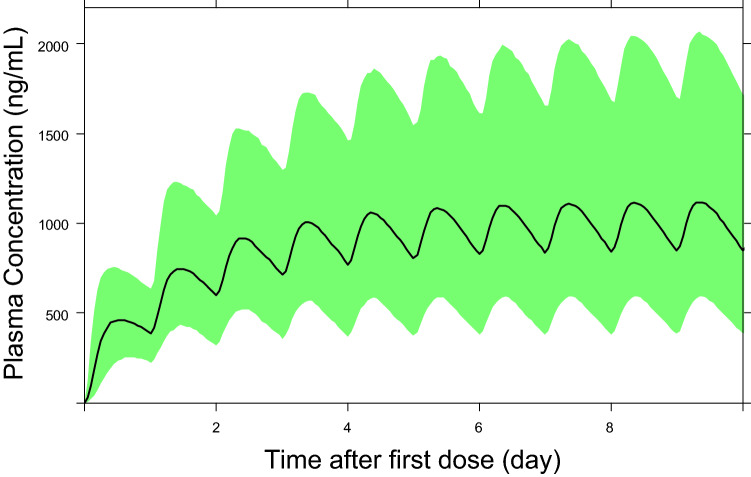
Simulation of tepotinib PK profile for a typical patient with NSCLC (59 years, 72 kg, serum albumin = 40 g/L, eGFR = 97.28 mL/min/1.73 m^2^, INR = 1.06) receiving 500 mg QD tepotinib TF3 with food. The solid black line represents the median prediction of the PK time profile, and the green shaded area represents a simulation-based 5–95% prediction interval for PK time profile. *eGFR* estimated glomerular filtration rate, *INR* international normalized ratio, *NSCLC* non-small cell lung cancer, *PK* pharmacokinetics, *QD* once daily, *TF* tablet formulation

In Fig. 3, the model-predicted time profile of tepotinib plasma concentration is shown for a typical patient with NSCLC (59 years, 72 kg, serum albumin = 40 g/L, eGFR = 97.28 mL/min/1.73 m^2^, INR = 1.06) receiving the clinical dose of 500 mg/day tepotinib TF3 with food.

**Fig. 4:**
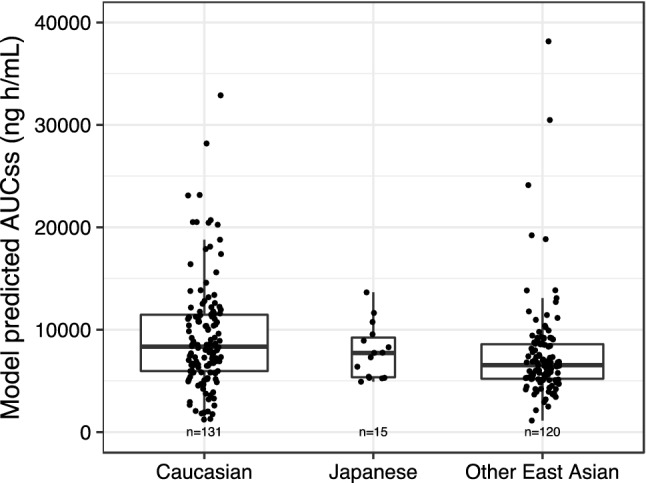
The distribution of tepotinib AUC_ss_ stratified by selected race categories, based on the final tepotinib population PK model and using the analysis data set. The predictions of AUC_τ,ss_ are for Caucasian, Other East Asian and Japanese participants in the tepotinib analysis data set receiving 500 mg tepotinib with the TF1 or TF2 formulation and having a standard breakfast. The horizontal line in the box indicates the median value, the box edges represent the 25th and 75th percentiles, and the whiskers extend from the box to the furthest data points still within a distance of 1.5 times the interquartile range from the box. Data points, which are jittered in the horizontal direction, show the individually predicted AUC_ss_ values. The numbers represent the number of individuals in each strata. *AUC*_*ss*_ area under the curve at steady state, *PK* pharmacokinetics, *TF* tablet formulation

The population PK analysis did not find ethnicity to be a statistically significant covariate on the exposure of tepotinib. This lack of influence was further investigated with boxplots comparing model-predicted AUC_τ,ss_ after 500 mg QD dosing for different ethnicity categories (Fig. 4), where the individual predicted AUC_τ,ss_ values also showed no trend.

**Fig. 5:**
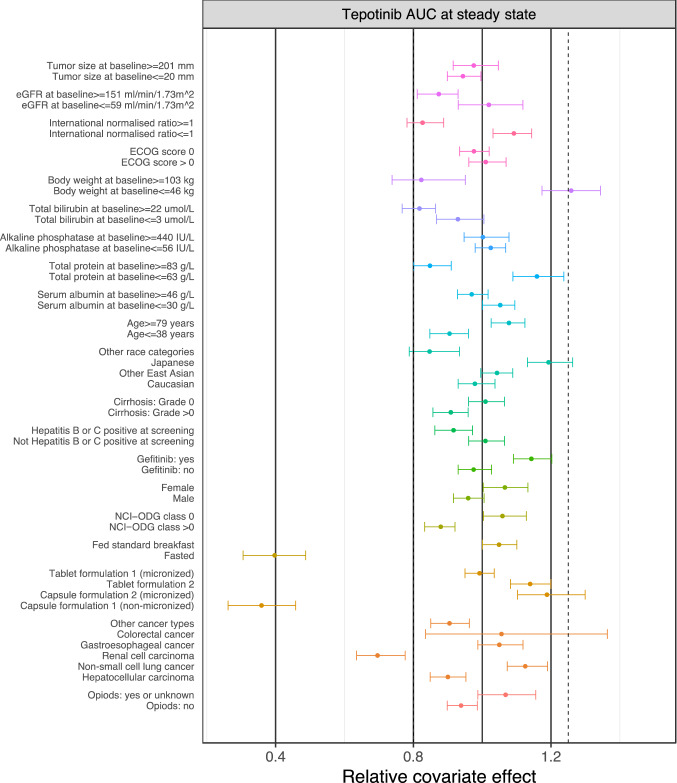
Forest plot showing the association of the predicted tepotinib AUC_ss_ and covariates assuming a dosing regimen of 500 mg tepotinib daily, based on the final tepotinib population PK model, for cancer patients in the analysis data set. The closed symbols represent the mean ratio of individual parameter estimates for the applicable covariate category or value (5th or 95th percentile for continuous covariates) percentile relative to the mean parameter estimate (vertical solid line) for cancer patients in the analysis dataset. The whiskers represent the 90% CI of the mean values, based on 100 bootstrap samples. *AUC*_*ss*_ area under the curve at steady state, *CI* confidence interval, *ECOG* Eastern Cooperative Oncology Group, *eGFR* estimated glomerular filtration rate, *NCI-ODG* National Cancer Institute Organ Dysfunction Group, *PK* pharmacokinetic

The influence of covariates on tepotinib and MSC2571109A AUC_τ,ss_ is illustrated in Fig. 5 and ESM 11. Most covariates showed no or marginal effects on tepotinib AUC with 90% CIs within the 80–125% range. These included race, hepatic impairment, renal impairment, age, sex, body weight, and intake of opioid analgesics or gefitinib. Exceptions included formulation, food intake status and tumor type of renal cell carcinoma.

The mean effect on MSC2571109A AUC_τ,ss_ mostly remained within the range of 80–125% for the typical patient with cancer in the analysis data set for all covariates, with the exception of moderate renal impairment, formulation, food intake status and tumor types (ESM 11). Given the negligible contribution of MSC2571109A to the clinical efficacy of tepotinib, it is unlikely that these covariate effects are clinically relevant.

## Discussion

Population PK modeling of tepotinib and MSC2571109A provides comprehensive model-based analytical tools to integrate both parent and metabolite PK profiles of 613 participants from 12 studies. These participants were administered four different formulations of tepotinib across a broad dose range of 30–1400 mg/day. These data have been well fitted to a two-compartment model with sequential zero- and first-order absorption and a first-order elimination from the central compartment for tepotinib, as well as a two-compartment model with input from the central compartment in the tepotinib model, scaled by FM and a first-order elimination from the central compartment for MSC2571109A. Since FM was unknown and could not be estimated from the data, it was fixed to 1 while the metabolite model was developed conditionally on the (fixed) tepotinib model. The fixed FM will only affect the numerical values of the primary metabolite PK parameters (CL and V) while the predicted metabolite concentrations and derived secondary parameters are unaffected. The model-predicted PK parameters are in agreement with the non-compartmental analyses of data from reported studies with tepotinib [8, 10, 14].

Tepotinib showed moderate PK IIV (33.5% for CL and 28.3% for bioavailability). No direct estimate of the intra-individual variability in tepotinib CL was obtained in the population PK analysis, but, given that the residual variability was 33.7% (which also includes variability due to the bioanalytical assay, potential model misspecification and any errors in correct documentation of PK sample timing), it may be concluded that the intra-individual variability in tepotinib PK is less than the IIV.

Tepotinib PK are approximately dose-proportional up to the therapeutic dose of 500 mg. At higher doses, tepotinib PK was shown to increase sub-proportionally to doses with limited exposure increases (less than a doubling) seen between 500 and 1400 mg. This finding should be interpreted with caution as most data for doses beyond 500 mg were obtained with the CF2 formulation in Study 001.

Tepotinib exhibits time-independent PK and has a long effective half-life of ~ 32 h with small peak-trough fluctuations, all of which support QD dosing. This differentiates tepotinib from other MET inhibitors, such as capmatinib, that require twice-daily dosing [20].

### Effect of intrinsic factors on tepotinib PK

#### Renal impairment

Mild and moderate renal impairment has been shown to have no clinically relevant effects on tepotinib PK (based on data from patients enrolled in the VISION study). In the covariate analysis, renal function was assessed using eGFR as determined by the MDRD formula. This metric was preferred over Cockcroft–Gault-predicted creatinine clearance as eGFR is independent of body weight and potentially leads to lower multi-collinearity with body mass covariates and less selection bias in the SCM procedure. The forest plot suggested that the apparent clearance of tepotinib did not show clinically meaningful association with eGFR: AUC_τ,ss_ was 2% higher in patients with moderate renal impairment (39.4 mL/min/1.73 m^2^ ≤ eGFR ≤ 59 mL/min/1.73 m^2^) in comparison to the mean observed eGFR value of 99.8 mL/min/1.73 m^2^. As expected, renal excretion minimally contributes to the total elimination: in the mass balance study, the majority (77.9%) of the total radiolabeled material was excreted via feces [14]. Urinary excretion was identified as a minor route with a recovery of only 13.6%. Both of these findings indicate that dose adjustment of tepotinib is not required for mild/moderate renal impairment.

#### Hepatic impairment

The effect of reduced liver function on F_par_ translates into lower total tepotinib AUC in participants with mild to moderate hepatic impairment (HI, based on NCI-ODWG classification), albeit to a not clinically relevant extent (90% CI for the mean ratio within the 80–125% interval, refer to Fig. 5). The effect of HI on exposure was specifically investigated in a hepatic impairment study (Study 0028) in which participants with moderate HI had 13% lower total plasma AUC, as determined by non-compartmental analysis, than healthy participants. However, in the same study, free tepotinib concentrations were similar in all hepatic function groups (manuscript in preparation).

Given the lack of clinically relevant changes in tepotinib free exposure and the flat exposure–response relationship over the exposure range achieved with the clinical dose of 500 mg/day [21], no loss of efficacy or elevated risk of adverse events is anticipated with mild/moderate hepatic impairment. Dose adjustment of tepotinib is not required for mild/moderate hepatic impairment, and there is insufficient evidence to draw conclusions with regard to severe hepatic impairment.

#### Ethnicity

Covariate model analysis and simulation-based comparisons suggested no impact of ethnicity on tepotinib PK. This finding reinforces the rationale of the VISION study as a multi-regional clinical trial (MRCT; conducted in the US, EU, Japan, China and other Asian countries) following ICH E17 principles [22], which could be accepted by regulatory authorities across regions and countries as the primary source of evidence to support marketing approval. This pivotal MRCT and the lack of ethnicity effect shown from the current modeling were important pillars in the first global approval in Japan.

#### Other intrinsic factors

There were no clinically relevant effects of age (> 18 years), sex, or body weight, hence no dose adjustments are required on the basis of any of these factors and a flat-dose regimen is supported by the analysis. Tumor type did not appear to influence tepotinib PK, and although lower exposures were seen in patients with renal cell carcinoma, this result must be considered in light of the low sample size (*N* = 5) for this patient group and the impact of other confounding factors.

### Extrinsic factors

#### Food and formulation

Tepotinib has been administered as several CFs and TFs during development. The CF1 formulation was the only one to use a non-micronized drug substance and was only used for the initial doses (up to 230 mg) in the first-in-human study [8]. Micronization of the drug substance in all other tested formulations was shown to increase tepotinib bioavailability, presumably via increased solubility leading to increased absorption. Likewise, the presence of food has been shown to increase tepotinib exposure due to increased in vivo solubility in the fed state. Studies in healthy participants confirmed these findings, showing an approximate two-fold increase in tepotinib exposure in the presence of food (high-fat meal) [11]. Tepotinib is, therefore, recommended to be administered with food to increase its bioavailability, and the phase 2 program, including the pivotal VISION trial, was conducted accordingly.

#### Co-administration with opioids or gefitinib

Opioid analgesics were included as continuous covariates based on the ability of µ-opioid receptor agonists to influence gastrointestinal motility. Twenty-four percent of the tepotinib observations were associated with concomitant administration of opioid analgesics. However, the observed effect of concomitant opioid use on CL/F did not lead to clinically meaningful changes in AUC.

Although, based on its ADME properties, no interaction with the EGFR inhibitor gefitinib was expected, concomitant administration of gefitinib was included as a covariate as patients in Study 006 received both drugs. Co-administration of tepotinib with gefitinib had no effect on tepotinib or gefitinib exposure [9].

## Conclusions

The PK of tepotinib and MSC2571109A were both well described using a two-compartment model with first-order elimination. Tepotinib has dose-dependent bioavailability above 500 mg and time-independent CL, with a profile appropriate for QD dosing.

The intrinsic factors of race, age, sex, body weight, mild/moderate hepatic impairment, and mild/moderate renal impairment, along with the extrinsic factors of opioid analgesic and gefitinib intake, had no relevant effect on tepotinib PK. Accordingly, no dose adjustment is required for any of these factors.

## Supplementary Information

Below is the link to the electronic supplementary material.

Supplementary file1 (DOCX 1934 kb)

## Data Availability

Any requests for data by qualified scientific and medical researchers for legitimate research purposes will be subject to Merck Healthcare KGaA, Darmstadt, Germany, Data Sharing Policy. All requests should be submitted in writing to Merck Healthcare KGaA, Darmstadt, Germany, data sharing portal, which can be found at https://www.merckgroup.com/en/research/our-approach-to-research-and-development/healthcare/clinical-trials/commitment-responsible-data-sharing.html. When Merck Healthcare KGaA, Darmstadt, Germany has a co-research, co-development, or co-marketing or co-promotional agreement, or when the product has been out-licensed, the responsibility for disclosure might be dependent on the agreement between parties. Under these circumstances, Merck Healthcare KGaA, Darmstadt, Germany will endeavor to gain agreement to share data in response to requests.
