# Supplementary material for: Population pharmacokinetic analysis of tepotinib, an oral MET kinase inhibitor, including data from the VISION study
Source: Cancer Chemother Pharmacol. 2022 Apr 6;89(5):655–69. doi: 10.1007/s00280-022-04423-5 (PMC9054876; doi:10.1007/s00280-022-04423-5)
Supplement: Supplementary file 1 — Supplementary file1 (DOCX 1934 kb) [file 280_2022_4423_MOESM1_ESM.docx]

**Electronic Supplementary Material**

ESM 1 Derivation of secondary parameters and illustration of covariate effects

For both tepotinib and MSC2571109A, secondary steady-state area under the curve (AUC_ss_) was derived from the final model results for Caucasian, Other East Asian and Japanese participants in the analysis data set, administered 500 mg daily as a tablet formulation with a standard meal. Results for these race categories were compared using box plots.

In addition, the following secondary pharmacokinetic (PK) parameters were derived for both tepotinib and MSC2571109A, for each unique combination of ID and the time-varying variables formulation, μ-opioids and dose: AUC_ss_, time to steady-state (T_ss_), maximum concentration at steady-state (C_max,ss_), trough concentration at steady-state (C_trough,ss_), volume of distribution at steady state (V_ss_), effective half-life (t_1/2eff_) and terminal half-life (t_1/2_).

The derivation of these secondary PK parameters is based on a bootstrap analysis (see following). Only participants with at least one observation above the lower limit of quantitation in the analysis data set were included in the derivation of the secondary parameters.

The association or lack of association between the observed covariates and both model-predicted parameters. Apparent clearance (CL) of parent drug (CL_par_), F_par_, apparent CL of metabolite (CL_met_) and secondary PK parameters (AUC_ss_, T_ss_, C_max,ss_ and C_trough,ss_) for cancer patients is illustrated using forest plots. The forest plots are also based on the bootstrap analysis.

In the bootstrap analysis, *N* = 100 new data sets (with the participants in the analysis data sets with at least one observation with the same number of participants as in the analysis data sets) were drawn with replacement from the analysis data sets. The sampling was stratified by study. The final models were fit to each of the resampled data sets. In the bootstrap for MSC2571109A, the parameter estimation was conditioned on the individual parameter estimates from the final tepotinib model.

For each of the *N* = 100 population parameter vectors, the *n* observed covariate vectors were used to predict the corresponding *n* primary and secondary parameters, where *n* = the unique combination of ID and the time-varying variables formulation, μ-opioids and dose (while National Cancer Institute Organ Dysfunction Group (NCI-ODG) class was handled as a time-varying covariate in the stepwise covariate method (SCM), its baseline value was used in the derivation of the forest plots since NCI-ODG class is derived from total bilirubin, alanine aminotransferase and aspartate aminotransferase, which were also assumed to be time-constant in the forest plot).

Descriptive statistics (mean, standard deviation and the 5^th^ and 95^th^ percentiles) were calculated for the *n* secondary parameters. These summary statistics are the bootstrap estimates of the typical individual value, standard error, and 90% confidence interval of the secondary parameters.

The data for the forest plots are based on the *N* = 100 population parameter vectors and the corresponding sets of *n* primary and secondary parameters:

1. For each of the categorical covariates:
   1. For each of the *N* = 100 population parameter vectors, compute the mean of the *n* primary and secondary parameters
   2. Compute the mean and the 5^th^ and 95^th^ percentiles of the *N* means to be used in the forest plots
2. For each of the continuous covariates:
   1. For each *N* = 100 population parameter vectors, compute the means of the parameter values that corresponds to the 0–5^th^ and 95–100^th^ percentile of the covariate
   2. Compute the mean and the 5^th^ and 95^th^ percentiles of the *N* = 100 means to be used in the forest plots
3. Use the mean of the *N* means with the precision given by the corresponding 5^th^ and 95^th^ percentile in the forest plots

The impact of the covariates in the forest plots are presented on a relative scale. The reference values are the arithmetic means of the *N* times *n* corresponding primary and secondary parameter values.

ESM 2 Descriptive statistics for the bootstrap predicted tepotinib typical individual PK parameters in cancer patients

| **Parameter** | **Mean** | **SE** | **5^th^ percentile** | **95^th^ percentile** |
| --- | --- | --- | --- | --- |
| CL_par_^a^ (L/h) | 19.3 | 0.6 | 18.3 | 20.2 |
| F_par_ actual doses | 1.12 | 0.04 | 1.06 | 1.18 |
| F_par_ 500 mg | 0.94 | 0.01 | 0.92 | 0.96 |
| F_par_ 250 mg | 1.04 | 0.02 | 0.99 | 1.08 |
| AUC_ss_ 500 mg (ng.h/mL) | 22,274 | 695 | 21,225 | 23,447 |
| AUC_ss_ 250 mg (ng.h/mL) | 12,277 | 397 | 11,701 | 12,914 |
| C_max, ss_ 500 mg (ng/mL) | 1,236 | 40 | 1,175 | 1,299 |
| C _max, ss_ 250 mg (ng/mL) | 683 | 33 | 627 | 737 |
| C_trough,ss_ 500 mg (ng/mL) | 952 | 34 | 903 | 1,008 |
| C_trough,ss_ 250 mg (ng/mL) | 523 | 26 | 481 | 557 |
| V_ss_^a^ (L) | 2,091 | 759 | 1,184 | 3,530 |
| T_ss_ (h) | 128.3 | 1.9 | 125.5 | 131.7 |
| t_1/2eff_ (h) | 32.1 | 0.5 | 31.4 | 32.9 |
| t_1/2_ (h) | 101.8 | 71.6 | 18.5 | 229.6 |

^a^ Multiplied by a factor of 0.9 to correct for the salt to base molar weight ratio.

*AUC_ss_* area under the curve at steady-state, *CL* apparent clearance, *C_max_* maximum concentration, C_max, ss_ maximum concentration at steady-state, *par* parent, *C_trough, ss_* trough concentration at steady-state, *eGFR* estimated glomerular filtration rate, *F* bioavailability, *INR* international normalized ratio, *NSCLC* non-small cell lung cancer, *PK* pharmacokinetics, *QD* once daily, *SE* standard error, *t_1/2_* terminal half-life, *t_1/2eff_* effective half-life, *TF* tablet formulation, *T_ss_* time to steady-state, V_ss_ volume of distribution at steady-state.

Note : The prediction is for a typical patient with NSCLC (59 years, 72 kg, serum albumin = 40 g/L, eGFR = 97.28 mL/min/1.73 m^2^, INR = 1.06) receiving 500 mg QD tepotinib TF3 with food.


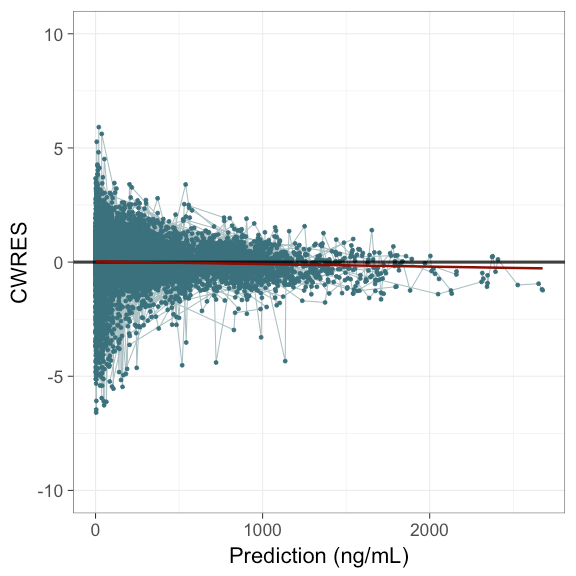

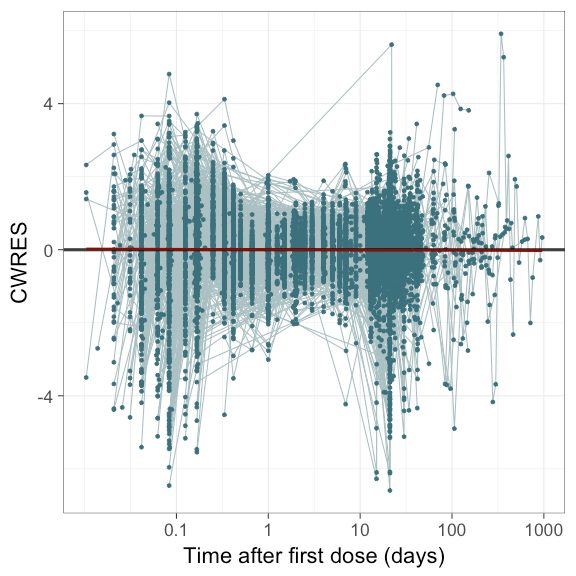


ESM 3 Conditional weight residuals (CWRES) versus population predicted tepotinib plasma concentrations, using the final tepotinib population pharmacokinetic model (linear and logarithmic scale).

Individual data points are indicated by dots and the points for each individual are connected with a line. The horizontal black line is the zero line and the red line is a smooth non-parametric trendline.


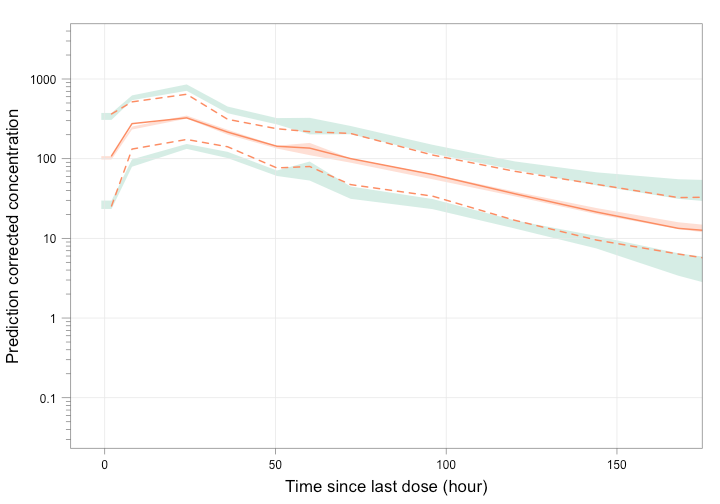


ESM 4 Prediction-corrected visual predictive checks of tepotinib concentrations versus time since last dose using the final tepotinib population pharmacokinetic model.

The data are presented on a log-linear scale. The solid and dashed red lines represent the observed median, 5^th^ and 95^th^ percentiles; the shaded red area represents the 95% confidence interval of the model predicted median and the shaded blue areas represent the 95% confidence interval of the model predicted 5^th^ and 95^th^ percentiles, based on 100 simulated data sets. The x-axis was cut at 175 hours.


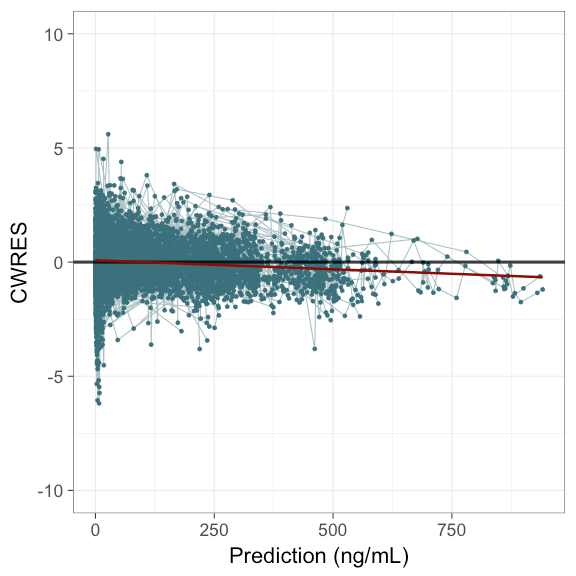


ESM 5 Conditional weight residuals (CWRES) versus population-predicted MSC2571109A plasma concentrations, using the final MSC2571109A population pharmacokinetic model.

Individual data points are indicated by dots and the points for each individual are connected with a line. The horizontal black line is the zero line and the red line is a smooth non-parametric trendline.


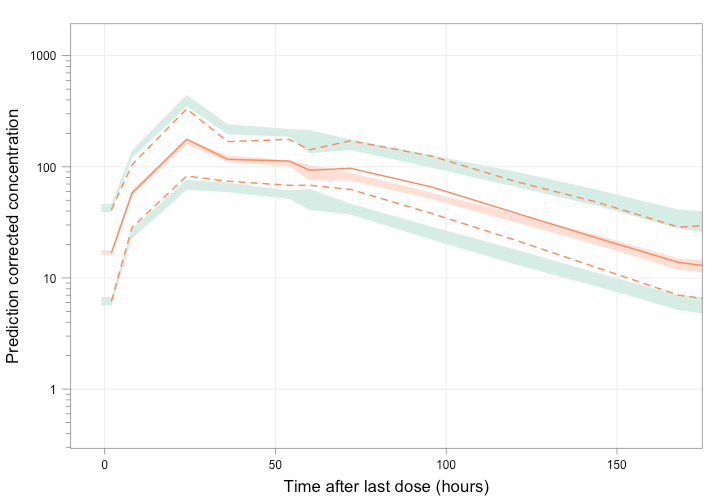


ESM 6 Prediction-corrected visual predictive checks of MSC2571109A concentrations versus time since last dose, using the final MSC2571109A population PK model.

The data are presented on a log-linear scale. The solid and dashed red lines represent the observed median, 5^th^ and 95^th^ percentiles; the shaded red area represents the 95% confidence interval of the model predicted median and the shaded blue areas represent the 95% confidence interval of the model predicted 5^th^ and 95^th^ percentiles, based on 100 simulated data sets. The x-axis was cut at 175 hours.


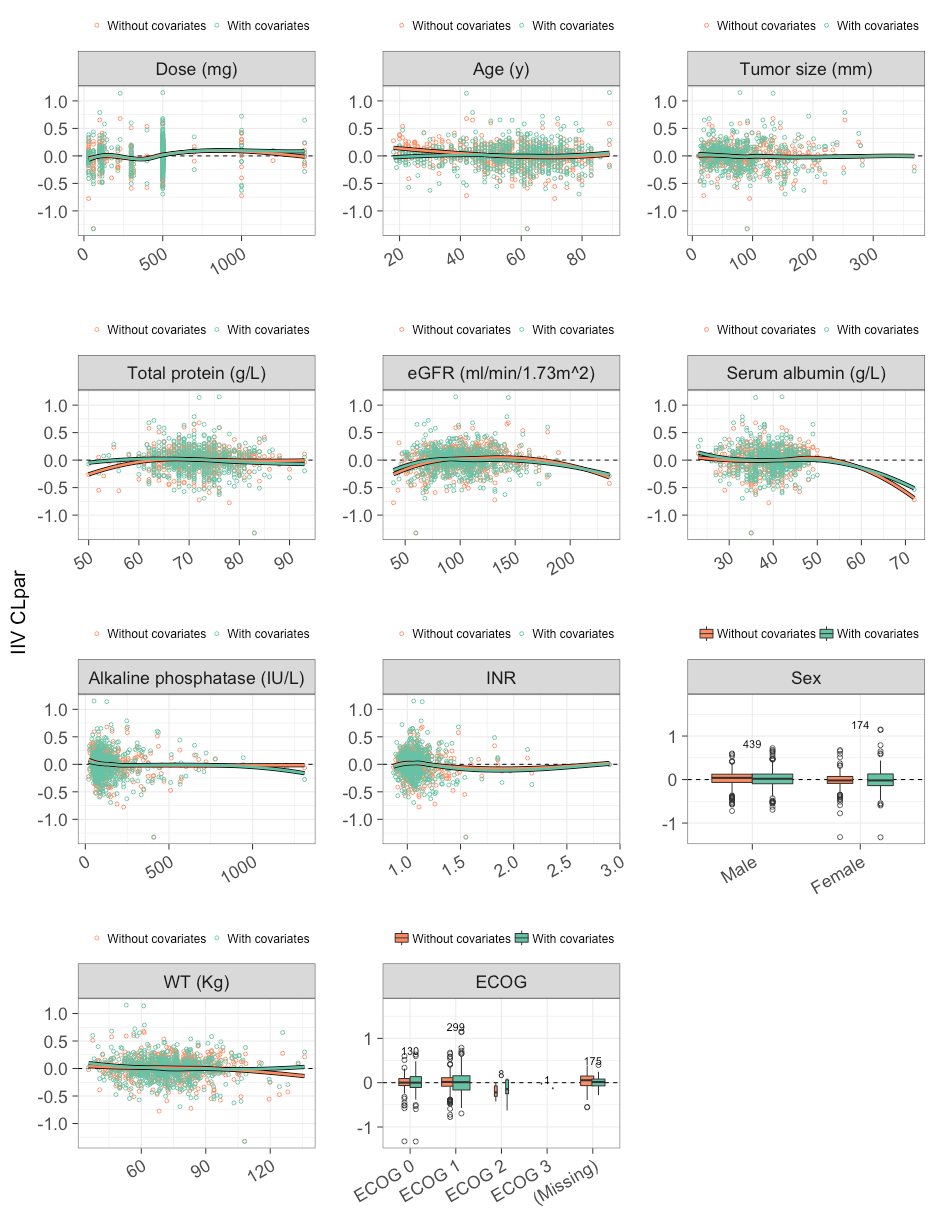


ESM 7 Distribution of tepotinib IIV in CL_par_ versus covariates before and after the addition of covariates to the model.

*CL* clearance, *ECOG* Eastern Cooperative Oncology Group, *eGFR* estimated glomerular filtration rate, *IIV* inter-individual variability, *INR* international normalized ratio, *par* parent, *WT* weight.


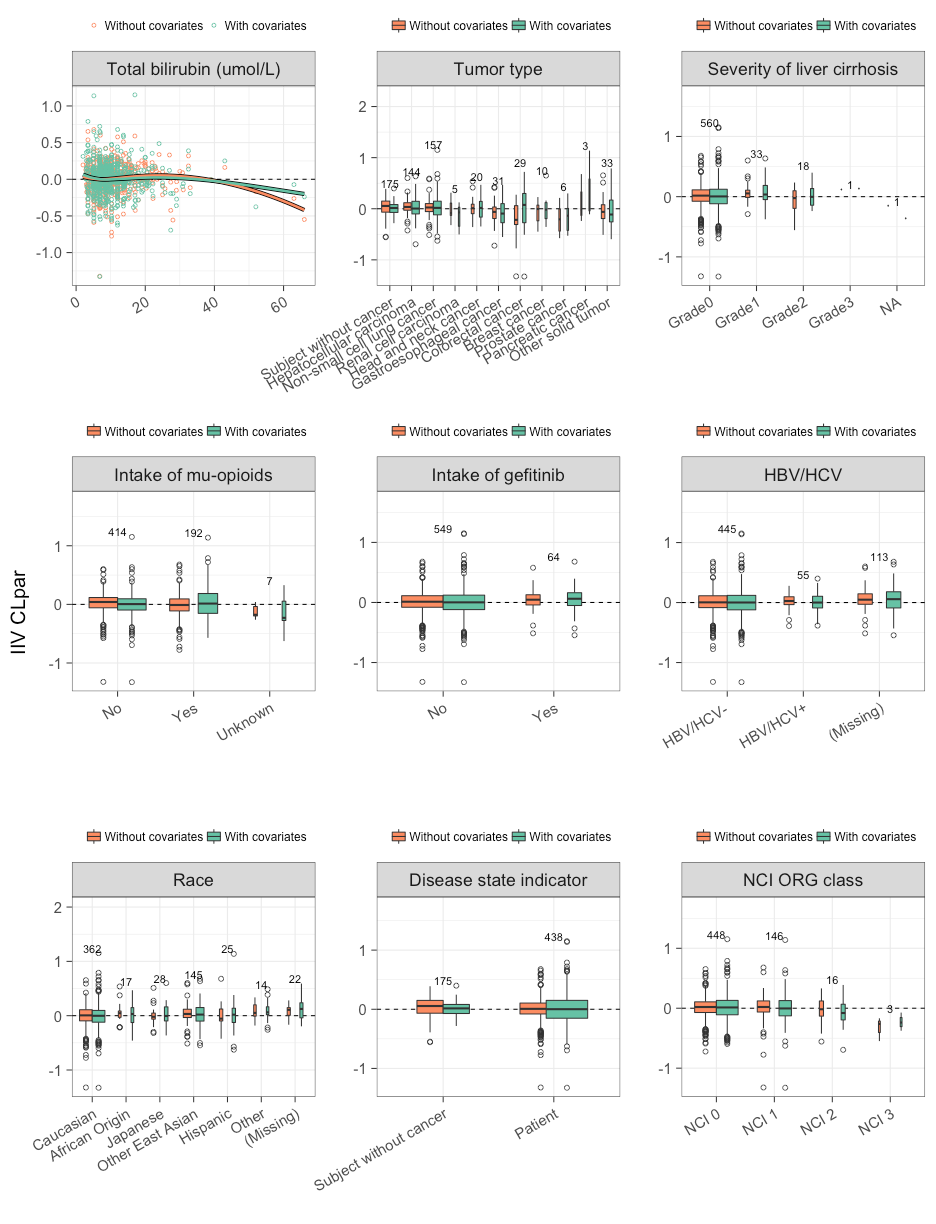


ESM 8 Distribution of tepotinib IIV in CL_par_ versus covariates before and after the addition of covariates to the model.

*CL* clearance, *HBV* hepatitis B virus, *HCV* hepatitis C virus, *IIV* inter-individual variability, *NCI* National Cancer Institute, *NCI ORG* National Cancer Institute Organ Dysfunction Group, *NCI* National Cancer Institute, *par* parent.


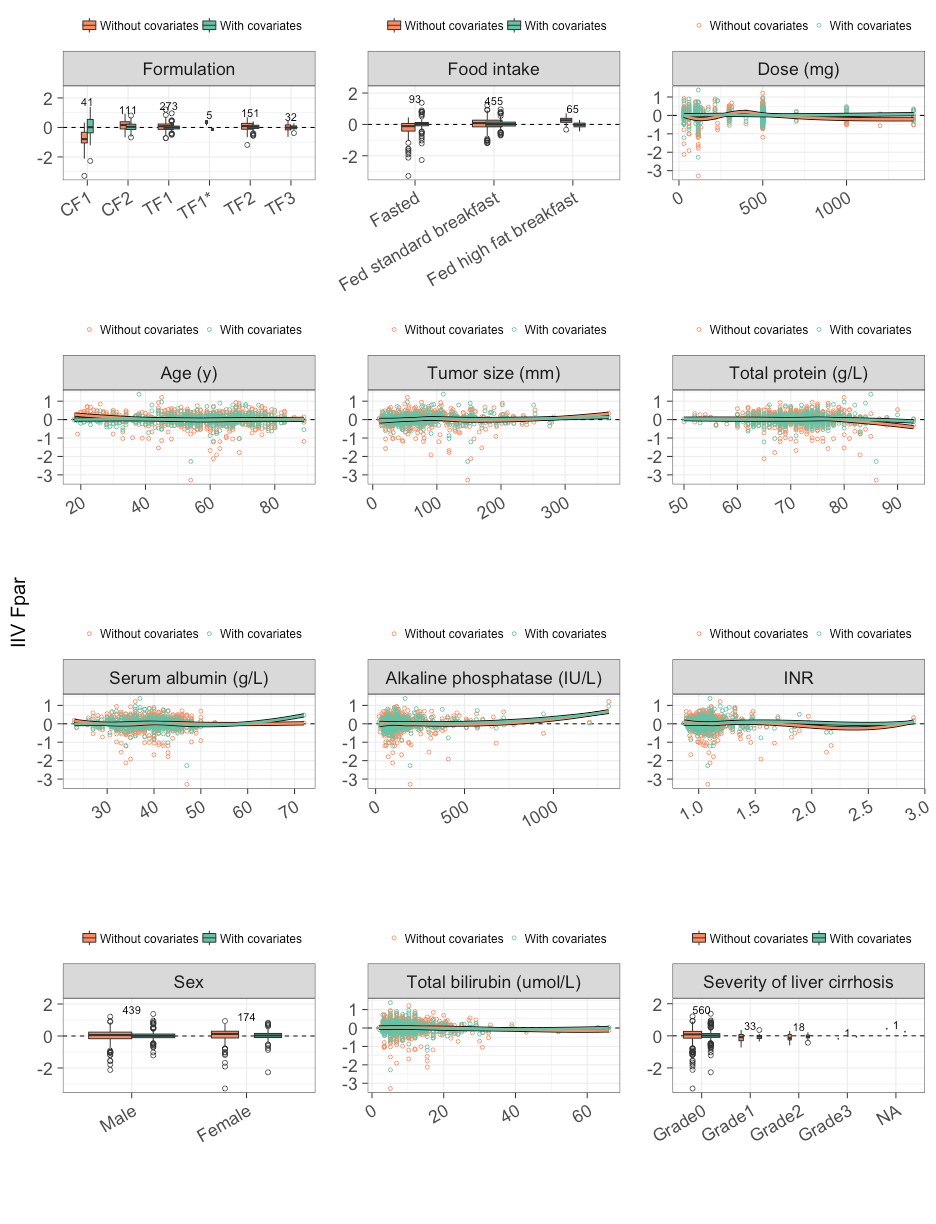


ESM 9 Distribution of tepotinib IIV in F_par_ versus covariates before and after the addition of covariates to the model.

*CF* capsule formulation, *F* bioavailability, *IIV* inter-individual variability, *INR* international normalized ratio, *NA* not applicable, *par* parent, *TF* tablet formulation.


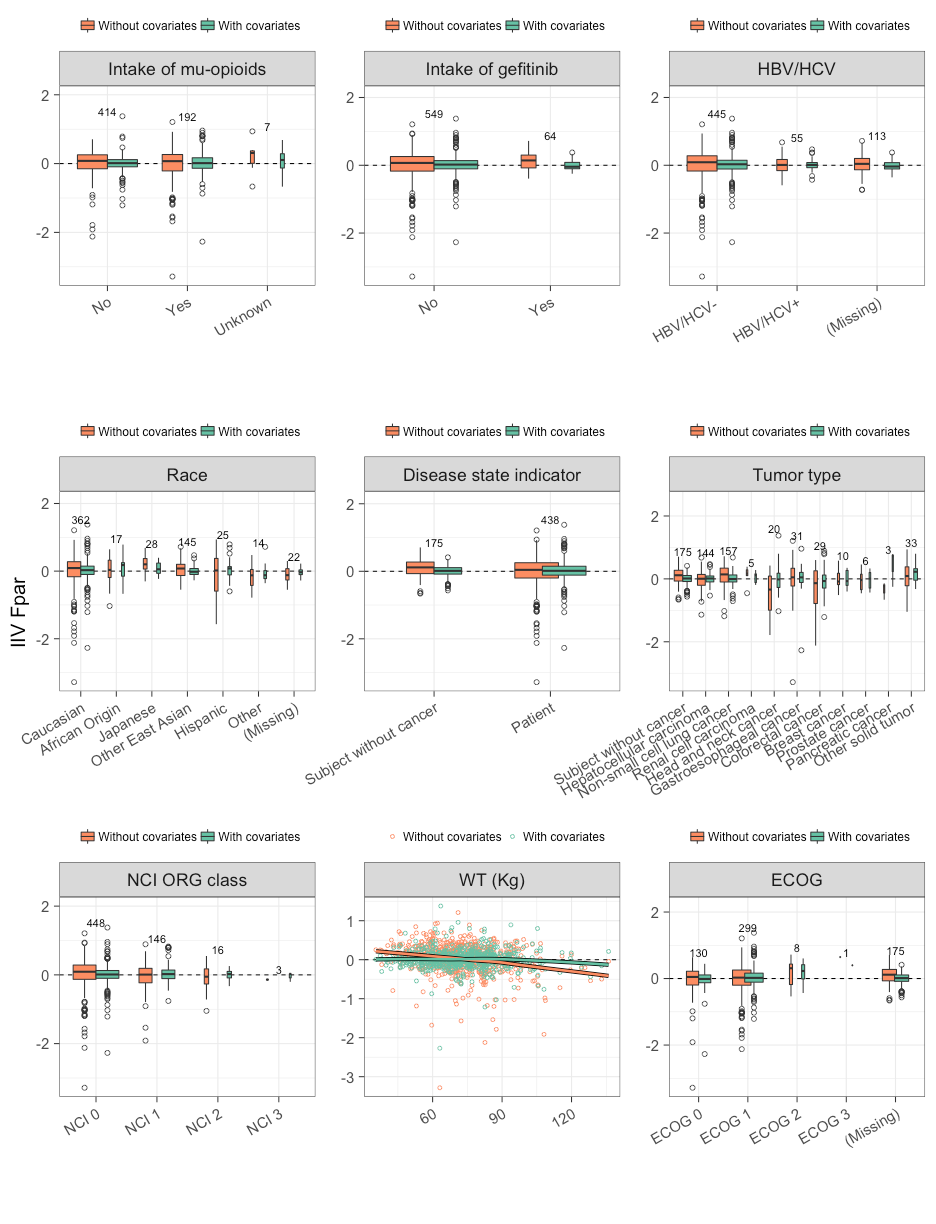


ESM 10 Distribution of tepotinib IIV in F_par_ versus covariates before and after the addition of covariates to the model.

*ECOG* Eastern Cooperative Oncology Group, *F* bioavailability, *HBV* hepatitis B virus, *HCV* hepatitis C virus, *IIV* inter-individual variability, *NCI* National Cancer Institute, *NCI ORG* National Cancer Institute Organ Dysfunction Group, *par* parent, *WT* wild-type.


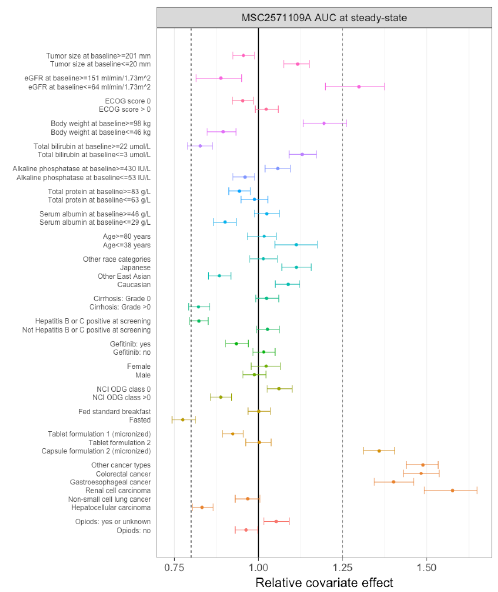


ESM 11 Forest plot showing the association of the predicted MSC2571109A AUC_ss_ and covariates assuming a dosing regimen of 500 mg tepotinib daily, based on the final MSC2571109A population PK model, for cancer patients in the analysis data set. *AUC_ss_* area under the curve at steady-state, *ECOG* Eastern Cooperative Oncology Group, *eGFR* estimated glomerular filtration rate, *NCI ODG* National Cancer Institute Organ Dysfunction Group, *PK* pharmacokinetics.


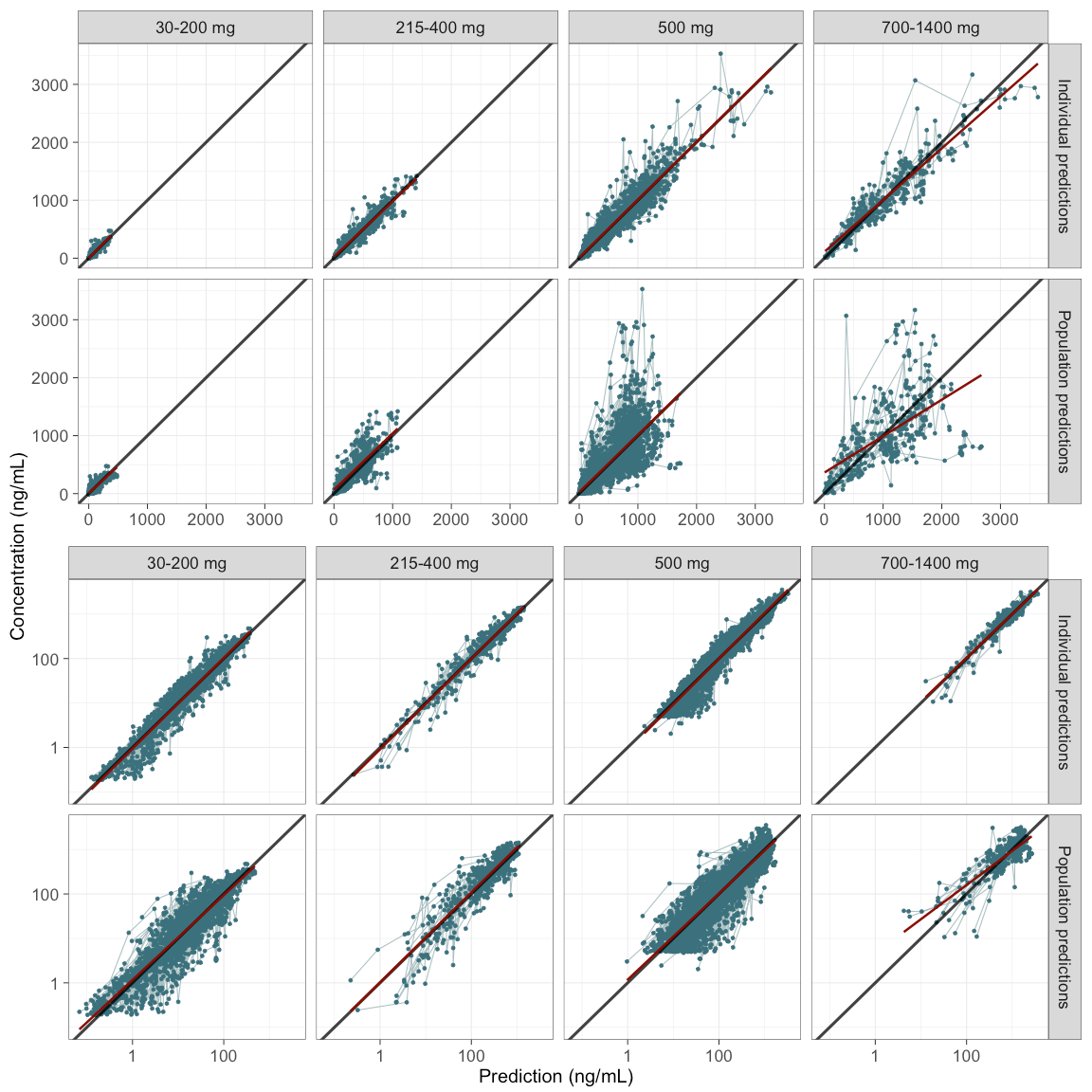


ESM 12 Observed versus predicted tepotinib plasma concentrations by dose ranges using the final tepotinib population PK model. Individual data points are indicated by dots and the points for each individual are connected with a line. Top set of panels using linear scales and the bottom set of panels use logarithmic scales. The black line is the line of unity and the red line is a smooth non-parametric trendline.


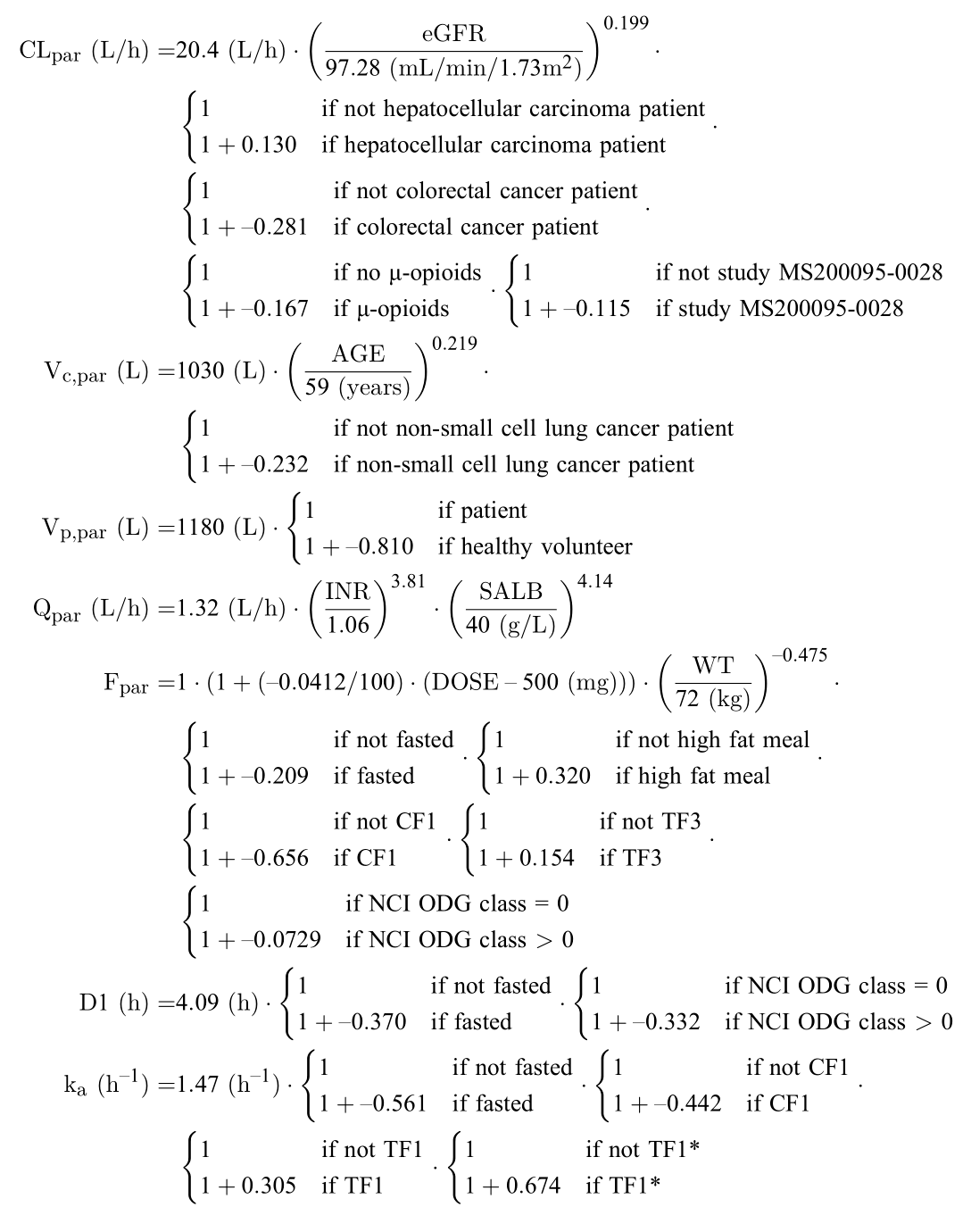


ESM 13 Equations for the final tepotinib covariate model.
